# Automatic Diagnosis of Bipolar Disorder Using Optical Coherence Tomography Data and Artificial Intelligence

**DOI:** 10.3390/jpm11080803

**Published:** 2021-08-18

**Authors:** Eva M. Sánchez-Morla, Juan L. Fuentes, Juan M. Miguel-Jiménez, Luciano Boquete, Miguel Ortiz, Elvira Orduna, María Satue, Elena Garcia-Martin

**Affiliations:** 1 Department of Psychiatry, Hospital 12 de Octubre Research Institute (i + 12), 28041 Madrid, Spain; emsmorla@gmail.com; 2 Faculty of Medicine, Complutense University of Madrid, 28040 Madrid, Spain; 3 CIBERSAM: Biomedical Research Networking Centre in Mental Health, 28029 Madrid, Spain; 4 Department of Ophthalmology, Miguel Servet University Hospital, 50009 Zaragoza, Spain; jlfuentesvernal@yahoo.es (J.L.F.); elvisabi14@hotmail.com (E.O.); mariasatue@gmail.com (M.S.); 5 Miguel Servet Ophthalmology Innovation and Research Group (GIMSO), Aragon Institute for Health Research (IIS Aragon), University of Zaragoza, 50009 Zaragoza, Spain; 6 Biomedical Engineering Group, Department of Electronics, University of Alcalá, 28801 Alcalá de Henares, Spain; jmanuel.miguel@uah.es (J.M.M.-J.); luciano.boquete@uah.es (L.B.); 7 Interdisciplinary Center for Security, Reliability and Trust (SnT), University of Luxembourg, 1855 Luxembourg, Luxembourg; migueloc41@gmail.com

**Keywords:** bipolar disorder, optical coherence tomography, neuroprogression, artificial intelligence

## Abstract

Background: The aim of this study is to explore an objective approach that aids the diagnosis of bipolar disorder (BD), based on optical coherence tomography (OCT) data which are analyzed using artificial intelligence. Methods: Structural analyses of nine layers of the retina were analyzed in 17 type I BD patients and 42 controls, according to the areas defined by the Early Treatment Diabetic Retinopathy Study (ETDRS) chart. The most discriminating variables made up the feature vector of several automatic classifiers: Gaussian Naive Bayes, K-nearest neighbors and support vector machines. Results: BD patients presented retinal thinning affecting most layers, compared to controls. The retinal thickness of the parafoveolar area showed a high capacity to discriminate BD subjects from healthy individuals, specifically for the ganglion cell (area under the curve (AUC) = 0.82) and internal plexiform (AUC = 0.83) layers. The best classifier showed an accuracy of 0.95 for classifying BD versus controls, using as variables of the feature vector the IPL (inner nasal region) and the INL (outer nasal and inner inferior regions) thickness. Conclusions: Our patients with BD present structural alterations in the retina, and artificial intelligence seem to be a useful tool in BD diagnosis, but larger studies are needed to confirm our findings.

## 1. Introduction

Bipolar disorder (BD) is a severe mental disorder that has a chronic or recurrent course characterized by high variability affecting its clinical manifestations, course, degree of functional deficit, and its neurobiological basis [1]. It is, therefore, a difficult disorder to diagnose, especially in the early stages of the disease, often resulting in delays in the initiation of adequate treatment [2,3]. Taking into account its marked heterogeneity, and the various clinical subtypes, it has, until now, been difficult to find biomarkers that could facilitate more accurate and timely diagnosis [4]. The development of objective and quantifiable diagnostic biomarkers is necessary to improve diagnosis and might also shed more light on the pathophysiology of the disease.

In the search for new biomarkers of brain structure and function, the study of alterations in the layers of the retina offers relevant information. The retina is an embryonic extension of the central nervous system (CNS), with which it shares some common features. It is connected to the CNS through the optic nerve and can provide an indirect assessment of inflammation and degeneration in the brain. The innermost layer of the retina is the retinal nerve fiber layer (RNFL), formed by the axons of the ganglion cells, which converge to form the optic nerve. The axons of these cells are not myelinated, so it is possible to study individual axons using imaging techniques such as optical coherence tomography (OCT) [5]. By considering the retina as part of the CNS, we can use it as a mirror to detect and establish relationships between neuronal changes that occur in the retina and those that occur in the brain. The OCT technique allows for development of biomarkers based on measurement of neuroretinal thickness, which is reproducible, reliable and quick to obtain [5,6,7,8,9].

In other neurological pathologies with degenerative etiology, such as multiple sclerosis, Alzheimer’s and Parkinson’s, OCT has been shown to be a reliable, inexpensive, and safe biomarker for diagnosis and follow-up [10,11,12].

In current BD research, evidence suggests that certain identified brain alterations could explain the existence of progressive changes associated with this disorder. The biological basis for clinical progression in BD is called neuroprogression [13]. Some biological alterations could be detected by structural studies of the retina using OCT [14].

To date, the results of neuroimaging studies of BD have indicated the presence of a diffuse pattern of brain alterations that include decreased volume of subcortical structures, cortical thinning, and alterations in white matter integrity [15]. The regions in which the loss of gray matter has been most consistently documented are those involved in the regulation of mood, i.e., the prefrontal area and the hippocampus. It has been observed that lithium appears to have a normalizing effect both in the alterations observed in the white matter and in the gray matter [16]. Conversely, other treatments, such as antiepileptic drugs, seem to have the opposite effect. The severity of symptoms and the number of manic episodes are also related to these structural changes [17,18]. Functional neuroimaging studies show alterations in frontal and temporal regions, including tracts that connect the prefrontal cortex with limbic subcortical areas, where the neural circuits would include those involved in emotion regulation and in the reward circuit [19]. These findings in neuroimaging studies could support the hypothesis of neuroprogression in BD.

Further to this, the findings of numerous studies measuring structural changes in the neuroretina associated with BD have been compiled in recent meta-analyses [20,21]. BD patients have shown abnormal OCT findings in the form of a decrease in full retinal thickness [22], thinning and/or volume reduction in the ganglion cell layer (GCL) [14,22,23,24], in the RNFL macular layer [14,23,24,25], and in the IPL (internal plexiform layer) and INL layers [14]. Analysis of the peripapillary retinal nerve fiber layer (pRNFL) also shows thinning in BD patients [14,22,26]. However, not all studies agree on the regions (quadrants, hemispheres) most affected, nor on the correlation with the time of development of the disease, or on other clinical variables [14,23,25,26,27,28,29,30]. These differences may be mainly due to the heterogeneity of the samples evaluated and methodological aspects of OCT [9].

Taking into account the difficulties in making an accurate and early diagnosis of BD based on clinical criteria and observations, the use of procedures based exclusively on data analysis, avoiding any type of a priori theoretical positioning, could have clear advantages.

Clinical decision-making based on data analysis using artificial intelligence (AI) techniques allows analysis of a multitude of variables (neurocognitive, neurophysiological, clinical, neuroimaging, genomic data, etc.) to obtain individual-level predictions. AI techniques, including Gaussian Naive Bayes, k-nearest neighbors algorithm (KNN), Decision Tree, Artificial Neural Networks and Support Vector Machine (SVM) have already been used to aid diagnosis in BD (see [31] for a review). More recent AI studies have used MRI [32] and genomic data [33], as well as neuroimaging and neuropsychological assessment [34], as variables. A recent review [35] focused on diagnosing BD by applying AI techniques to neuroimaging analysis.

To the authors’ knowledge, there are no studies that apply AI techniques to the analysis of OCT reports in BD. The purpose of this paper is to describe the characteristic structural retinal changes revealed by OCT in patients with BD and to apply AI methods to identify objective and quantifiable markers to improve the diagnosis of BD using this safe, comfortable, non-invasive, and cost-effective test.

## 2. Materials and Methods

Seventeen individuals diagnosed with BD type I and forty-two gender- and age-matched healthy controls were included in the study. They were recruited at the Department of Psychiatry of Miguel Servet University Hospital. All procedures adhered to the tenets of the Declaration of Helsinki, which has been approved by the ethics committee (institutional review board approval was obtained for this study), and all participants gave written informed consent to participate in the study. 

The minimum sample size needed to find significant differences in OCT between the healthy and the patient group was calculated. Based on a preliminary study performed by us [14], a minimum sample size was calculated to detect differences of at least 2 μm in the RNFL or GCL thickness measured by OCT, applying a bilateral test with α = 5% risk and β = 10% risk (i.e., with 90% power). In order to obtain a sufficient sample of patients with bipolar disorder to allow an in-depth study of the natural history of the disease, the unexposed/exposed ratio was determined to be 0.5. From these data it was concluded that at least 18 eyes would be needed in each group. We included 18 eyes in the group of subjects with bipolar disorder, but one was excluded due to poor scan quality. Forty-two control eyes were included to increase the power of the study.

The diagnosis of BD was established by a trained psychiatrist and was based on the Diagnostic and Statistical Manual of Mental Disorders, 5th edition (DSM-V) criteria (American Psychiatric Association, 2013, Washington, DC, United States of America).

Inclusion criteria were confirmed BD diagnosis, best-corrected visual acuity (BCVA) of 20/50 or higher (using a Snellen chart, +0.4 in LogMar) in each eye to allow performance of the protocol, and intraocular pressure of less than 21 mmHg. Exclusion criteria were the presence of significant refractive errors (>5 diopters of spherical equivalent refraction or 3 diopters of astigmatism), intraocular pressure ≥21 mmHg; media opacifications, concomitant ocular diseases, including history of glaucoma or retinal pathology, substance abuse or dependence, and systemic conditions (diabetes, cardiovascular pathology) that could affect the visual system.

The healthy controls presented no history or evidence of ocular, psychiatric or neurological disease of any nature and their BCVA was 20/50 or higher (using a Snellen chart, +0.4 in LogMar). The control subjects met the same exclusion criteria as the patients. One eye per subject was randomly selected and included in the study.

We performed prior analysis to ensure that both groups in the study had comparable smoking habits in order to avoid bias as a confounding factor. To do this, we stratified the sample into four groups: Group 0: non-smokers, or ex-smokers who gave up more than 5 years ago; Group 1: smokers who smoked 0 to 10 cigarettes a day, or ex-smokers who gave up fewer than 5 years ago; Group 2: smokers who smoked 10 to 20 cigarettes a day; Group 3: smokers who smoked more than 20 cigarettes a day. The distribution was compared to confirm there were no significant differences between the two groups before the start of the analytical statistical study. Both groups have been adjusted for the number of cigarettes consumed per day.

**Figure 1:**
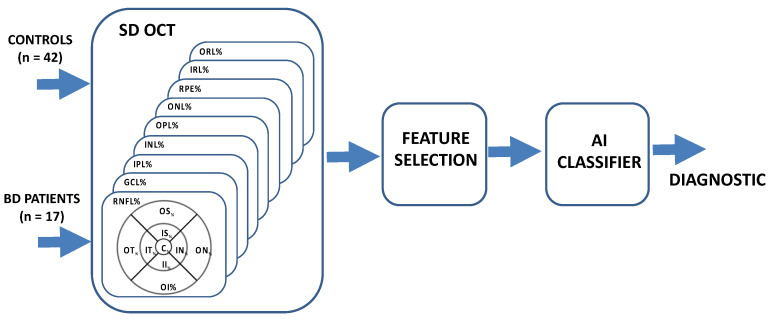
General block diagram. RNFL: retinal nerve fiber layer, GCL: ganglion cell layer, IPL: inner plexiform layer, INL: inner nuclear layer, OPL: outer plexiform layer, ONL: outer nuclear layer, RPE: retinal pigment epithelium, IRL: inner retinal layer, ORL: outer retinal layer. ETDRS chart: C: central, IN: inner nasal, ON: outer nasal, IS: inner superior, OS: outer superior, IT: inner temporal, OT: outer temporal, II: inner inferior, OI: outer inferior, AI: artificial intelligence.

A block diagram of the system implemented is shown in Figure 1. The retinal thicknesses were obtained with a SPECTRALIS HRA+OCT device (Heidelberg Engineering, Heidelberg, Germany), which works with a scan rate of 40 kHz, scan depth of 1.8 mm, 7 μm axial resolution (3.5 μm/pixel) and 14 μm lateral resolution. Using built-in software (Heidelberg Eye Explorer, version 1.9.10.), measurements of the RNFL (retinal nerve fiber layer), GCL (ganglion cell layer), IPL (inner plexiform layer), INL (inner nuclear layer), OPL (outer plexiform layer), ONL (outer nuclear layer), RPE (retinal pigment epithelium), IRL (inner retinal layer) and ORL (outer retinal layer) were obtained in the 9 regions defined by the ETDRS chart: Central (C), Inner Nasal (IN), Outer Nasal (ON), Inner Superior (IS), Outer superior (OS), Inner Temporal (IT), Outer Temporal (OT), Inner Inferior (II) and Outer Inferior (OI).

### Statistical Analysis

Values were expressed as mean values ± standard deviation (±SD) for normally distributed variables and as median and quartiles (median [interquartile range]) for non-normally distributed variables.

The statistical tests used for the data analysis were for continuous variables: the Shapiro–Wilk test (analysis of normative distribution), t-Student (difference between means in normative distribution) and U-Man (difference between medians in non-normative distribution). For the analysis of differences in categorical variables, the χ^2^-test (chi-square test) was used. *p* values < 0.05 were considered statistically significant.

Associations between OCT variables were investigated using Pearson’s correlation coefficient. The AUC (Area Under the receiver operating Curve) has been used for the analysis of the discriminant capacity of the variables; a variable is considered to have discriminant capacity between controls and BD if AUC > 0.75. The classification process was summarized with accuracy and AUC parameters.

Statistical analyses were performed using IBM SPSS Statistics 25 software (SPSS Inc., Chicago, IL, USA). 

## 3. Results

Sociodemographic and clinical characteristics of the sample are shown in Table 1. There were no significant differences in age (*p* = 0.703), gender (*p* = 0.45), or left or right eye numbers (*p* = 0.48) between the control and BD groups. The duration of the disease in years was 20.64 ± 6.48 and the BD was diagnosed at 30.00 ± 13.84 (years).

There were no significant differences between groups as regards intraocular pressure (IOP) or smoking status (9 of the individuals in the BD group—52.94%—were current smokers (or had been in the past 5 years)); 23 of the controls—54.76%—were smokers [or had been in the past 5 years] (*p* = 0.132)]. Pharmacological status revealed that 14 patients were being treated with lithium (82.3%), 2 with valproate (11.8%), and 1 currently received no treatment (5.9%).

Table 2 shows the results of the structural study carried out in the 9 segmented layers and in the 9 ETDRS regions. In each case, the thickness value, the *p* value between controls and patients, and the discriminant capacity for each variable are indicated and were evaluated using AUC.

In most cases, the thickness of the retinal layers in healthy subjects was greater than the thickness of the layers in BD patients.

In the RNFL layer this difference was not significant in any region while in the OT region the retinal thickness in the BD group was greater than in the controls. In all the regions of the GCL layer the retinal thickness of the controls exceeded that of the BD patients, the differences being significant in the 4 regions that define the inner ring (IN, IS, IT, II). In the IPL layer, the retinal thickness in controls was significantly greater in the IN and II regions. In the INL layer, the thickness of the BD patients’ retinal layers significantly exceeded that of the controls, except in the central region.

In the OPL layer, significant differences in retinal thickness between controls and patients were observed in the OT region, in the IRL layer, in the IN region and, finally, in the ORL layer in the OS region.

### 3.1. Correlations between the Most Discriminating Variables

**Figure 2:**
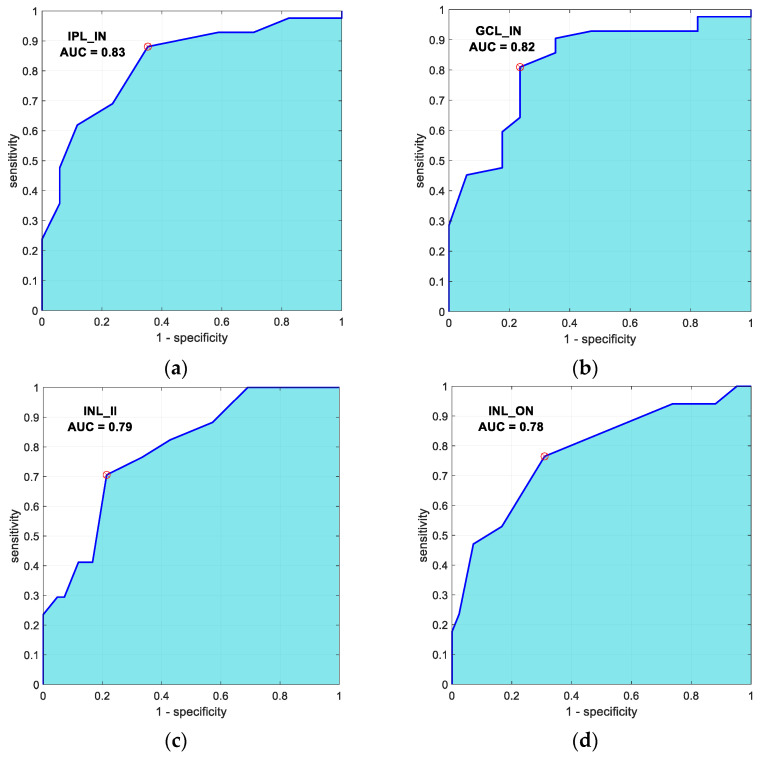
ROC curves of the 4 optical coherence tomography variables with greatest discriminant capacity (control subjects vs. bipolar disorder patients). (**a**) Inner Plexiform layer-inner nasal (AUC = 0.83); (**b**) Ganglion cell layer-inner nasal (AUC = 0.82); (**c**) Inner nuclear layer- inner inferior (AUC = 0.79); (**d**) Inner nuclear layer-outer nasal (AUC = 0.78).

The variables presented in Table 2 with the highest discriminant capacity (AUC > 0.75) between controls and patients are (layer_region) IPL_IN (AUC = 0.83), GCL_IN (AUC = 0.82), INL_II (AUC = 0.79) and INL_ON (AUC = 0.78). The AUC curves of these four variables are shown in Figure 2.

Table 3 shows the correlations between these 4 variables. The significant correlations are (GCL_IN vs. IPL_IN = 0.938, *p* < 0.01) (information between the variables GCL_IN and IPL_IN is highly redundant), (GCL_IN vs. INL_II = 0.36, *p* = 0.005), (IPL_IN vs. INL_II = 0.46, *p* < 0.01) and (INL_ON vs. INL_II = 0.63, *p* < 0.01). 

### 3.2. Correlation with Disease Duration, Number of Hospitalizations and Manic Episodes

In order to evaluate the association between structural changes in the retina in BD and the duration and clinical course of the disease, we evaluate the Pearson correlation between the four variables with most discriminant diagnostic capacity, disease duration, number of hospitalizations, and number of manic episodes of the patients. 

We found no significant association with disease duration: r (GCL_IN vs. ID): −0.034 (*p* = 0.90), r (IPL_IN vs. ID): +0.009 (*p* = 0.975), r_(INL_ON vs. ID): +0.368 (*p* = 0.196) and r_(INL_II vs. ID): −0.037 (*p* = 0.89). 

No association was found between retinal involvement and the number of hospitalizations, but we did find a slight significant correlation between INL_ON thickness and the number of manic episodes (r = 0.66, *p* = 0.02). 

### 3.3. Automatic Classification

In order to evaluate the performance of the diagnostic aid system, multiple automatic classifiers have been tested, available in the Classification Learner App from Matlab^®^ ver. R2020a (MathWorks, Natick, MA, USA). For each of the classifiers, all the possible combinations of the 4 input features with the highest AUC have been tested, and the combination that obtains the best result has been selected. The leave-one-out cross validation method was used in all experiments. Cross validation is a technique used to evaluate the results of an automatic classifier and guarantees independence between training and test data. In the leave-one-out validation, every input is in turn used to test the model induced from the other inputs [36].

Because the Classification Learner App supports a maximum number of input examples = 50, and in our case it is (42 + 17), the functions generated for each classifier were exported to the Matlab workspace and modified to be able to apply the leave-one-out validation to our database.

The following classifiers have been tested: Gaussian Naive Bayes, KNN (distance metrics: Medium, Cubic, Cosine, Weighted) and SVM (linear, quadratic, and Gaussian kernels).

The Gaussian Naive Bayes classification algorithm assigns the label of the class that maximizes the posterior probability of each input, with the “naive” assumption of independence between input features and a normal distribution [37]. Given an instance to be classified, represented by a vector ***x*** = (*x*_1_, ..., *x*_n_), it assigns probabilities *P*(*C_k_*∣*x*_1_, ..., *x*_n_) for each *k* possible classes *C_k_*. By Bayes’ theorem, the conditional probability is:

(1) $$\left(C_{i}|x\right)=\frac{P\left(x|C_{i}\right)\cdot P\left(C_{i}\right)}{P\left(x\right)}$$

where $P\left(C_{i}|x\right)\;$ is the posterior probability that ***x*** belongs to class $C_{i}$; $P\left(x|C_{i}\right)\;$ is the likelihood function (conditional probability that a data point  x belongs to class $\;C_{i}$) and $\;P\left(C_{i}\right)$ is the prior probability.

The KNN algorithm calculates a similarity measurement or distance between a new entry and the set of entries used as the training set; the tested sample is assigned to the class of its nearest neighbor [38]. In KNN, *k* indicates the number of nearest neighbors to be considered in decision making. The distance between the test sample and the training set may be identified by different metrics [39]: Euclidean or L2 norm ($D\left(x,y\right)=\sqrt[{2}]{\sum_{i=1}^{n}{\left|x_{i}-y_{i}\right|}^{2}}$), cosine (difference in direction between two vectors), cubic ($D\left(x,y\right)=\sqrt[{3}]{\sum_{i=1}^{n}{\left|x_{i}-y_{i}\right|}^{3}}$), etc. The weighted distance between two *n*-dimensional vectors ***x*** and ***y*** is: $D\left(x,y\right)=\sqrt[{2}]{\sum_{i=1}^{n}w_{i}\cdot \;{\left|x_{i}-y_{i}\right|}^{2}}$), where $\sum_{i=1}^{n}w_{i}=1$.

In a two-class learning task, an SVM looks for the hyperplane that separates two different classes with maximum margin (support vectors). If the input feature is not linearly separable, a non-linear transformation may be performed to get a higher dimensional space using a kernel function to improve the separability between the two classes in the new space. Kernel functions may be linear (non tunable parameters), polynomial (($1+x_{i}^{T}\cdot x_{j}{)}^{p}$) or Gaussian ($\exp(-\gamma \|x_{i}-x_{j}\|{}^{2})$), tunable parameter: width of the function (γ). The penalization of misclassified examples can then be controlled using the box constraint parameter or soft-margin penalty (C) [40]. 

Table 4 shows the accuracy (ratio of correct predictions to total predictions) and AUC results for several of the experimental classifiers, showing for each classifier the input features that obtain the best result. These results indicate good diagnostic capacity in the available database, since in all cases the accuracy value is equal to or greater than 0.87 and the AUC value is greater than or equal to 0.90.

Table 5 shows the confusion matrix for the classifier with the best results (Linear SVM: accuracy = 0.95, AUC = 0.97), using the variables IPL_IN, INL_ON and INL_II as inputs.

## 4. Discussion

In this study, it was found that the retinal thickness in patients diagnosed with bipolar disorder presents abnormalities in comparison with healthy volunteers. It was also observed that there was no difference between both groups in RNFL thickness. In the CGL layer there was a significant difference in regions IN (*p* = 0.00), IS (*p* = 0.021), IT (*p* = 0.020) and II (*p* = 0.014). In the IPL layer the differences were present in regions IN (*p* = 0.00) and II (*p* = 0.035). The regions affected in the INL layer were IN (*p* = 0.041), ON (*p* = 0.001), IS (*p* = 0.009), OS (*p* = 0.012), IT (*p* = 0.017), OT (*p* = 0.042), II (*p* = 0.001) and OI (*p* = 0.004). In the OPL, IRL and ORL layers the regions affected were OT (*p* = 0.020), IN (*p* = 0.009) and OS (*p* = 0.013), respectively. It was also observed that there was no difference between both groups in RNFL, ONL and RPE thickness.

The results of previous OCT studies in BD detected an overall decrease in the thickness of the RNFL (predominantly in the temporal sectors), as well as a thinning of the CCG, which have been inversely correlated in cross-sectional studies with the duration of the disease, number of hospitalizations and number of manic episodes [14,23,25,27]. These data support the idea of neuroprogression or neurological loss [41], understood as a process of pathological reorganization of the central nervous system [42], possibly related to an increase in inflammatory and oxidative activity, occurring during relapses [43], and therefore capable of causing a gradual reduction in the thickness of the retina. Additionally, the OCT technique makes it possible to obtain meaningful differences between images in a short period of time [44], which would be of interest in longitudinal studies of BD in order to establish the neurological structural effects on the brain of affective episodes [43,45], as has been suggested in patients with BD with a predominance of manic polarity [46]. Interestingly, we found a slight association between number of manic episodes and OCT abnormalities, which might suggest that BD has a neuroprogressive component, at least in a subset of BD patients with manic polarity [43,45,46].

As pointed out earlier, in other neurodegenerative diseases such as multiple sclerosis and Alzheimer’s disease, the decrease in macular thicknesses is more striking than the results obtained in our study. Retinal thickness abnormalities were also recently described in psychiatric pathologies or chronic mental diseases, such as schizophrenia [21,47]. Researchers have detected retinal layer thinning, that is consistent with the classic gray- and white-matter atrophy observed on neuroimaging in these pathologies, suggesting that OCT may be a useful biomarker tool in studying the neurobiology of psychosis.

It is thought that the impairment of the neuroretina in BD, in addition to being more gradual than in other neurodegenerative diseases, could be slowed by treatment with lithium, which may have a neuroprotective effect, minimizing or slowing neuronal degeneration [48]. However, correlation study of OCT thicknesses and lithium treatment duration did not reveal significant associations in our population; methodological issues, especially related to the small sample size in the BD group, could explain this lack of association.

There is great variability in the clinical picture, which suggests heterogeneity among the BD subjects included in the studies. This heterogeneity makes it difficult to interpret the different results available. Therefore, a longitudinal study with years of follow-up, with a larger sample size that includes other clinical subtypes of BD, and with a correlation analysis between drug treatment, number of affective episodes, and RNFL degeneration could provide more solid data on this topic.

In this paper, an objective method to aid diagnosis of BD has been developed based on the OCT measurements of both BD patients and controls and analysis of these data using artificial intelligence techniques. It was found that different types of automatic classifiers obtain very similar results, which reinforces the idea that the classification is robust.

AI has been used previously in diagnosis of BD, but using other types of patient information. A recent review [31] reported AUCs of 0.698 for structural magnetic resonance imaging (sMRI), 0.754 for functional magnetic resonance imaging (fMRI) and 0.712 for both combined. The papers included in this review include data from structural and functional neuroimaging, genetic, EEG features, neuropsychological tests and serum biomarkers, but do not include structural data from the retina for the diagnosis of BD. Results in this study were slightly better than other automatic diagnostic systems using smart classifiers, but with other input variables. In [41], using MRI and an SVM classifier the authors found an AUC of 0.71 in differentiating BD from controls. More recently, [34], also using an SVM classifier, obtained an accuracy of 87.60% considering neuroimaging and neurocognitive measures as input. The results of these lines of study suggest the benefit of undertaking automatic diagnostic studies of BD in which data from OCT, neuroimaging and neurocognitive studies, among others, are considered as inputs. Tests with a larger and multicenter database will allow us to elucidate which classifier or set of classifiers allow the best diagnosis to be achieved [49].

One of the main limitations of this study is that the sample size was small. In addition, the results only refer to patients with BD type I; therefore they cannot be generalized to the whole BD spectrum. Another limitation is that the BD patients in our study were heavy smokers and progressive retinal changes in the smoking population have been widely demonstrated. Smokers present a significant decrease in the peripapillary RNFL, foveal thickness, and the ganglion cell complex [26,50]. To ascertain that the differences found in the retinal measurements between BD patients and controls in our study were not caused by smoking we carefully analyzed our samples and established that the number of smokers was not significantly different between the groups. Another limitation was the fact that all patients were receiving pharmacological treatment. It cannot be dismissed that pharmacological treatment, most especially mood stabilizers, may significantly influence OCT measurements. Lithium has been found to have a neuroprotective role in patients with bipolar disorder [51], and valproate may show protection against GCL loss [52]. Most of the patients in our sample (82.3%) were treated with lithium and therefore a comparative subgroup analysis of the possible role of treatments was not possible in our study due to the small sample size. Finally, the fact that it is a cross-sectional design constitutes a limitation in itself. Despite these limitations, our population is representative of the BD type I population, and comparable with the healthy group, since there are no significant differences in the possible confounding factors (IOP, age, gender, smoking status, systemic diseases affecting OCT measurements) between the two study groups, and therefore the results found can be extrapolated and valid. These findings open a new diagnostic pathway in a pathology in which the diagnosis is currently and eminently clinical and there is a lack of objective tests, such as OCT, which in addition to being useful for diagnosis, as suggested by our study, also allows axonal damage to be quantified on a regular basis. This helps to monitor the course of the disease and can also be repeated as many times as desired because of its non-invasive nature. Additionally, this test can even be performed in non-hospital health centers with non-specialized personnel and can be easily visualized and interpreted by the neurologist, psychiatrist or primary care physician. This helps to resolve geographic barriers and also to monitor the pathology in patients in whom going to a hospital center is a risk (for example, subjects with multiple pathologies or those at high risk in case of contagion by COVID-19) or in subjects for whom travel presents difficulties (e.g., limited mobility, rural residence, geographic barriers, etc.).

In conclusion, the BD patients evaluated in our study present structural alterations in the retina. Our results suggest that the use of AI techniques to classify retinal thickness data, obtained by OCT, can help in the diagnosis of BD. In our opinion, it is necessary to continue this line of research, and in new future directions, including other measures in the automatic classification obtained in multicenter studies, with larger samples followed up during the course of the disease, including the early stages, with the aim of improving diagnostic precision in BD and identifying additional biological markers of clinical progression that might explain the progressive changes associated with this disorder, at least in a subset of bipolar patients. Early detection and understanding biological changes in clinical progression might help to identify therapeutic targets to promote better outcomes in BD.

## Figures and Tables

**Table 1:** Sociodemographic and clinical characteristics of the sample.

	Controls *n* = 42	BD *n* = 17	*p* Value
Mean age (years ± SD)	49.74 ± 17.01	51.47 ± 11.94	*p* (t-test) = 0.703
Male:Female ratio	13:29	7:10	χ^2^(1) = 0.56, *p* = 0.45
Number of R:L eyes analyzed	18:24	9:8	χ^2^(1) = 0.49, *p* = 0.48
Duration of disease (years ± SD)	--	20.64 ± 6.48	--
Age when disease diagnosed (years ± SD)	--	30.00 ± 13.84	--
Number of hospitalizations	--	2 [3.0]	--
Number of manic episodes	--	8.33 ± 4.22	--

Abbreviations: BD, bipolar disorder; SD, standard deviation; R, right; L, left.

**Table 2:** Thicknesses in the 9 layers of the retina and in the 9 ETDRS regions. Mean ± SD (μm) (normal distribution, Shapiro–Wilk test *p* ≥ 0.05). Median [interquartile range] (μm) (non-normal distribution, Shapiro–Wilk test *p* < 0.05).

Layer	RNFL		GCL		IPL		INL		OPL		ONL		RPE		IRL		ORL	
	C	BD	C	BD	C	BD	C	BD	C	BD	C	BD	C	BD	C	BD	C	BD
Central	12.0 [3.0]	11.65 ± 2.09	16.62 ± 3.78	14.0 [5.5]	21.26 ± 3.51	20.12 ± 4.86	20.76 ± 6.26	18.0 [10.0]	25.71 ± 5.60	23.0 [8.5]	95.0 [16.25]	92.0 [15.0]	16.0 [2.0]	15.0 [2.0]	189.45 ± 25.03	185.06 ± 20.48	90.0 [5.0]	88.18 ± 4.59
	*p* (U-test) = 0.14 AUC = 0.62		*p* (U-test) = 0.17 AUC = 0.61		*p* (t-test) = 0.32 AUC = 0.61		*p* (U-test) = 0.86 AUC = 0.51		*p* (U-test) = 0.47 AUC = 0.56		*p* (U-test) = 0.46 AUC = 0.56		*p* (U-test) = 0.056 AUC = 0.66		*p* (t-test) = 0.52 AUC = 0.58		*p* (U-test) = 0.28 AUC = 0.59	
Inner Nasal (IN)	22.21 ± 2.88	20.47 ± 3.78	53.0 [6.25]	44.71 ± 6.94	43.0 [4.25]	38.59 ± 4.12	40.50 [5.0]	43.47 ± 3.73	32.0 [7.0]	35.47 ± 8.46	76.0 [16.25]	70.94 ± 16.15	15.05 ± 1.62	15.0 [3.5]	267.0 [25.0]	254.06 ± 15.31	82.50 ± 3.09	81.82 ± 3.09
	*p* (t-test) = 0.06 AUC = 0.68		*p* (U-test) = 0.00 AUC = 0.82		*p* (U-test) = 0.00 AUC = 0.83		*p* (U-test) = 0.041 AUC = 0.67 ^#^		*p* (U-test) = 0.56 AUC = 0.55 ^#^		*p* (U-test) = 0.79 AUC = 0.52		*p* (U-test) = 0.71 AUC = 0.53 ^#^		*p* (U-test) = 0.009 AUC = 0.72		*p* (t-test) = 0.45 AUC = 0.56	
Outer Nasal (ON)	51.90 ± 7.99	44 [18]	38.00 [6.0]	36.82 ± 5.08	30.0 [3.25]	29.35 ± 3.46	34.0 [4.0]	37.12 ± 3.39	28.0 [4.25]	29.0 [4.0]	55.81 ± 7.98	55.18 ± 9.63	13.0 [2.0]	13.00 ± 1.77	239.0 [18.75]	239.29 ± 11.27	78.95 ± 2.84	77.71 ± 2.78
	*p* (U-test) = 0.18 AUC = 0.61		*p* (U-test) = 0.62 AUC = 0.54		*p* (U-test) = 0.91 AUC = 0.51		*p* (U-test) = 0.001 AUC = 0.78 ^#^		*p* (U-test) = 0.055 AUC = 0.66 ^#^		*p* (t-test) = 0.79 AUC = 0.52		*p* (U-test) = 0.66 AUC = 0.54		*p* (U-test) = 0.87 AUC = 0.51 ^#^		*p* (t-test) = 0.13 AUC = 0.62	
Inner Superior (IS)	25.07 ± 2.62	24.0 [3.5]	52.0 [5.25]	51.00 [7.0]	42.0 [4.0]	40.06 ± 3.36	40.0 [4.5]	43.29 ± 2.69	31.0 [6.0]	30.0 [11.0]	73.50 [11.0]	69.94 ± 11.80	16.0 [3.0]	16.0 [3.0]	264.0 [23.75]	263.0 [17.5]	82.0 [3.5]	81.0 [3.5]
	*p* (U-test) = 0.12 AUC = 0.63		*p* (U-test) = 0.021 AUC = 0.69		*p* (U-test) = 0.051 AUC = 0.66		*p* (U-test) = 0.009 AUC = 0.72 ^#^		*p* (U-test) = 0.83 AUC = 0.52		*p* (U-test) = 0.65 AUC = 0.54		*p* (U-test) = 0.97 AUC = 0.50		*p* (U-test) = 0.46 AUC = 0.56		*p* (U-test) = 0.14 AUC = 0.62	
Outer superior (OS)	39.57 ± 5.56	38.0 [.8.0]	36.0 [5.0]	33.88 ± 3.66	29.0 [3.25]	27.76 ± 2.61	31.83 ± 2.58	33.82 ± 2.83	25.0 [3.0]	26.0 [3.0]	61.45 ± 8.55	59.12 ± 6.70	14.0 [2.25]	13.06 ± 1.30	225.5 [15.25]	229.0 [19.0]	80.00 ± 2.78	78.00 ± 2.47
	*p* (U-test) = 0.50 AUC = 0.56		*p* (U-test) = 0.16 AUC = 0.62		*p* (U-test) = 0.13 AUC = 0.63		*p* (t-test) = 0.012 AUC = −0.69 ^#^		*p* (U-test) = 0.56 AUC = 0.55 ^#^		*p* (t-test) = 0.32 AUC = 0.58		*p* (U-test) = 0.18 AUC = 0.61		*p* (U-test) = 0.70 AUC = 0.53		*p* (t-test) = 0.013 AUC = 0.70	
Inner Temporal (IT)	17.0 [3.0]	17.06 ± 1.68	47.50 [6.5]	44.0 [11.0]	41.50 [4.25]	40.0 [7.5]	37.0 [5.0]	40.47 ± 4.53	29.5 [5.0]	31.0 [3.5]	73.50 [9.0]	71.35 ± 10.71	14.0 [3.0]	15.0 [3.0]	250.5 [21.0]	246.0 [18.5]	82.0 [4.25]	80.94 ± 3.34
	*p* (U-test) = 0.36 AUC = 0.57		*p* (U-test) = 0.020 AUC = 0.69		*p* (U-test) = 0.18 AUC = 0.61		*p* (U-test) = 0.017 AUC= 0.70 ^#^		*p* (U-test) = 0.28 AUC = 0.59 ^#^		*p* (U-test) = 0.32 AUC = 0.58		*p* (U-test) = 0.82 AUC = 0.52		*p* (U-test) = 0.20 AUC = 0.61		*p* (U-test) = 0.24 AUC = 0.60	
Outer Temporal (OT)	19.0 [2.0]	20.0 [3.5]	37.5 [7.0]	35.0 [6.5]	32.50 [3.0]	32.12 ± 3.59	33.50 [4.25]	34.94 ± 2.08	26.5 [2.25.]	28.65 ± 2.71	57.57 ± 7.36	54.94 ± 7.06	13.0 [1.0]	12.24 ± 1.09	208.0 [15.25]	207.0 [19.5]	78.40 ± 2.67	77.06 ± 2.05
	*p* (U-test) = 0.43 AUC= 0.56 ^#^		*p* (U-test) = 0.27 AUC = 0.59		*p* (U-test) = 0.57 AUC = 0.55		*p* (U-test) = 0.042 AUC = 0. 67 ^#^		*p* (U-test) = 0.020 AUC = 0. 69 ^#^		*p* (t-test) = 0.21 AUC = 0.60		*p* (U-test) = 0.27 AUC = 0.59		*p* (U-test) = 0.72 AUC = 0.53 ^#^		*p* (t-test) = 0.067 AUC = 0.65	
Inner Inferior (II)	26.62 ± 4.36	24.76 ± 4.24	53.0 [6.0]	50.0 [8.0]	41.0 [4.0]	39.0 [6.5]	41.0 [4.0]	45.47 ± 4.16	34.57 ± 8.54	37.47 ± 8.54	63.95 ± 11.69	61.12 ± 15.81	14.43 ± 1.65	14.29 ± 1.45	262.5 [19.5]	259.0 [18.5]	80.02 ± 2.78	79.41 ± 2.74
	*p* (t-test) = 0.14 AUC = 0.64		*p* (U-test) = 0.014 AUC = 0.70		*p* (U-test) = 0.035 AUC = 0.67		*p* (U-test) = 0.001 AUC = 0 79 ^#^		*p* (t-test) = 0.24 AUC = 0.59 ^#^		*p* (t-test) = 0.51 AUC = 0.53		*p* (t-test) = 0.77 AUC = 0.52		*p* (U-test) = 0.20 AUC = 0.61		*p* (t-test) = 0.44 AUC = 0.56	
Outer Inferior (OI)	41.07 ± 7.83	38.82 ± 9.19	31.50 [5.25]	32.0 [4.0]	26.0 [4.0]	26.35 ± 2.32	31.0 [3.0]	32.94 ± 2.11	27.0 [5.0]	27.59 ± 2.55	50.83 ± 6.98	49.35 ± 7.75	12.50 [1.25]	12.0 [2.0]	208.5 [18.5]	208.0 [15.5]	76.95 ± 2.95	76.41 ± 2.87
	*p* (t-test) = 0.35 AUC = 0.62		*p* (U-test) = 0.84 AUC = 0.52 ^#^		*p* (U-test) = 0.73 AUC = 0.53		*p* (U-test) = 0.004 AUC = 0.74 ^#^		*p* (U-test) = 0.29 AUC = 0.58 ^#^		*p* (t-test) = 0.48 AUC = 0.53		*p* (U-test) = 0.38 AUC = 0.57		*p* (U-test) = 0.79 AUC = 0.52		*p* (t-test) = 0.52 AUC = 0.54	

Abbreviations: T-test: Student’s t test, U-test: Mann–Whitney U test, AUC: area under the receiver operation curve, ^#^: the values in BD exceed the values of the controls. BD, bipolar disorder; C: controls; RNFL, retinal nerve fiber layer; GCL, ganglion cell layer; IPL, inner plexiform layer; INL, inner nuclear layer; OPL, outer plexiform layer; ONL, outer nuclear layer; RPE, retinal pigment epithelium; IRL, inner retinal layer; ORL, outer retinal layer.

**Table 3:** Pearson correlation between the four discriminant variables (*n* = 59, controls and patients). Abbreviations: GCL, ganglion cell layer; IPL, inner plexiform layer; INL, inner nuclear layer; IN, inner nasal; ON, outer nasal; II, inner inferior.

	GCL_IN	IPL_IN	INL_ON	INL_II
GCL_IN	1	0.938 *p* < 0.01	0.184 *p* = 0.16	0.36 *p* = 0.005
IPL_IN	--	1	0.25 *p* = 0.056	0.46 *p* < 0.01
INL_ON	--	--	1	0.63 *p* < 0.01
INL_II	--	--	--	1

**Table 4:** Results with several classifiers. Accuracy and AUC results for several of the experimental classifiers, showing for each classifier the input features that obtain the best result.

Classifier	Input Features				Accuracy	AUC
	GCL_IN	IPL_IN	INL_ON	INL_II		
Gaussian Naive Bayes	X	X	X	X	0.89	0.91
KNN (k = 3, Euclidean)	X		X	X	0.92	0.95
KNN (k = 3, Cubic)	X		X	X	0.92	0.95
KNN (k = 3, Cosine)		X	X	X	0.89	0.95
KNN (k = 3, Weighted)		X	X	X	0.89	0.92
SVM (Linear, C = 2)		X	X	X	0.95	0.97
SVM (Quadratic, *p* = 2, C = 2)		X		X	0.89	0.90
SVM ($\gamma =\sqrt{3}$, C = 2)	X		X	X	0.87	0.92
SVM ($\gamma =4\sqrt{2}$, C = 2)	X			X	0.92	0.92

Abbreviations: GCL, ganglion cell layer; IPL, inner plexiform layer; INL, inner nuclear layer; IN, inner nasal; ON, outer nasal; II, inner inferior; AUC, area under the curve.

**Table 5:** Confusion matrix. Classifier: Linear SVM with inputs IPL_IN, INL_ON and INL_II.

	Predicted Control	Predicted BD
Actual control	40	2
Actual BD	1	16

Abbreviations: IPL, inner plexiform layer; INL, inner nuclear layer; IN, inner nasal; ON, outer nasal; II, inner inferior.

## Data Availability

The data presented in this study are available from the corresponding author upon reasonable request.
